# Using a daily monitoring system to reduce treatment position override rates in external beam radiation therapy

**DOI:** 10.1002/acm2.13629

**Published:** 2022-05-04

**Authors:** Naichang Yu, Anthony Magnelli, Danielle LaHurd, Anthony Mastroianni, Eric Murray, Mike Close, Brian Hugebeck, John H. Suh, Ping Xia

**Affiliations:** ^1^ Department of Radiation Oncology Taussig Cancer Institute Cleveland Clinic Cleveland Ohio USA

**Keywords:** automation, external beam radiotherapy, patient safety, treatment couch overrides, treatment positioning errors

## Abstract

**Purpose/objectives**: To report our 7‐year experience with a daily monitoring system to significantly reduce couch position overrides and errors in patient treatment positioning.

**Materials and methods**: Treatment couch position override data were extracted from a radiation oncology–specific electronic medical record system from 2012 to 2018. During this period, we took several actions to reduce couch position overrides, including reducing the number of tolerance tables from 18 to 6, tightening tolerance limits, enforcing time outs, documenting reasons for overrides, and timely reviewing of overrides made from previous treatment day. The tolerance tables included treatment categories for head and neck (HN) (with/without cone beam CT [CBCT]), body (with/without CBCT), stereotactic body radiotherapy (SBRT), and clinical setup for electron beams. For the same time period, we also reported treatment positioning–related incidents that were recorded in our departmental incident report system. To verify our tolerance limits, we further examined couch shifts after daily kilovoltage CBCT (kV‐CBCT) for the patients treated from 2018 to 2021.

**Results**: From 2012 to 2018, the override rate decreased from 11.2% to 1.6%/year, whereas the number of fractions treated in the department increased by 23%. The annual patient positioning error rate was also reduced from 0.019% in 2012, to 0.004% in 2017 and 0% in 2018. For patients treated under daily kV‐CBCT guidance from 2018 to 2021, the applied couch shifts after imaging registration that exceeded the tolerance limits were low, <1% for HN, <1.2% for body, and <2.6% for SBRT.

**Conclusions**: The daily monitoring system, which enables a timely review of overrides, significantly reduced the number of treatment couch position overrides and ultimately resulted in a decrease in treatment positioning errors. For patients treated with daily kV‐CBCT guidance, couch position shifts after CBCT image guidance demonstrated a low rate of exceeding the set tolerance.

## 1 INTRODUCTION

Secondary to ongoing technological advancements in radiation oncology, radiation therapy continues to evolve as an effective, personalized, and precise treatment modality for many cancer patients. Given the increasing complexity of radiation therapy delivery and the recognition of limitations of the conventional equipment‐centered quality assurance (QA) program, a QA process that is both patient‐ and process‐centered has been advocated.^1^ After the New York Time articles^2^, ^3^ reported radiation oncology safety issues in 2010, additional reports from single institutional experiences^4^, ^5^, ^6^, ^7^, ^8^ have demonstrated that process flaws and human errors occur more frequently than equipment malfunction. Process‐centered QA focuses on quality controls (QCs) of the process to ensure the desirable outcomes. The process of radiotherapy can be divided into three major steps: simulation, treatment planning, and treatment delivery. For the entire process of radiotherapy, QCs for treatment delivery are rarely published. Although the introduction of image‐guided radiotherapy (IGRT), especially kilovoltage cone‐beam CT (kV‐CBCT), has significantly improved treatment positioning precision, IGRT is only applicable to approximately 60% of patients and is not entirely error‐proof.

Incident learning, quantitative quality measures, and process consistency (Six Sigma) are the principles for process QCs that have been developed in other industries.^9^, ^10^, ^11^, ^12^ In radiation oncology, incident learning systems have been established in national and single institutional levels.^6^, ^7^, ^8^, ^13^ These systems have successfully identified the failure modes and weaknesses of QA programs in radiation processes. Root cause analyses of medical incidents in our department revealed that treatment positioning errors were the most frequent cause of treatment incidents and that treatment couch parameter overrides were the primary culprit even with imaging guidance. For example, one incident occurred in our department was due to accidentally dragging the aligned CBCT images off after imagine registration, resulting in unintended misalignment. Couch position consistency control could prevent such mistakes by alerting therapists that the couch position was outside of tolerance. It should be noted that a consistent couch position alone is not sufficient to ensure a correct treatment position. However, a couch position that is outside of tolerance may indicate an incorrect treatment position. The controlling couch position is the first layer of QC in patient positioning and imagining guidance is the second layer of QC in patient positioning and treatment target localization. During the past 7 years, we took multiple actions to reduce treatment couch position overrides. The purpose of this study is to report our results in reducing couch position overrides and to correlate this reduction with the number of treatment positioning errors.

## 2 MATERIALS AND METHODS

### 2.1 Immobilization and treatment positioning

At our main campus, we treat an average of 140–150 patients/day with external beam photon radiation using six linear accelerators (Linacs). In 2017, we replaced our treatment machines from multiple vendors with those from a single vendor, all equipped with kV‐CBCT, and installed three of the six Linacs with six degree of freedom (6DoF) couches. Table 1 lists the immobilization devices used in our department, along with clinical examples. These devices were indexed to the treatment couch so that daily variations in treatment couch positions could be controlled, particularly the vertical and longitudinal positions. For patients treated for thorax and pelvis tumors, couch position variations in the lateral direction are controlled by positioning these patients in a head hold, which is indexed to the treatment couch in a designed location.

**TABLE 1:** Typical Immobilization devices (manufacturers) used in our department

Immobilization device	Clinical examples	Manufacture
Routine immobilization system	HN, breast, pelvis, thorax, prostate	Orfit, Jericho, NY
Abdominal compression (with full‐body bag below)	SBRT lung, SBRT liver	CIVCO, Orange City, IA
Full body bag	SBRT spine, SBRT pancreas SBRT prostate	BodyFix, Elekta, Stockholm, Sweden

**FIGURE 1:**
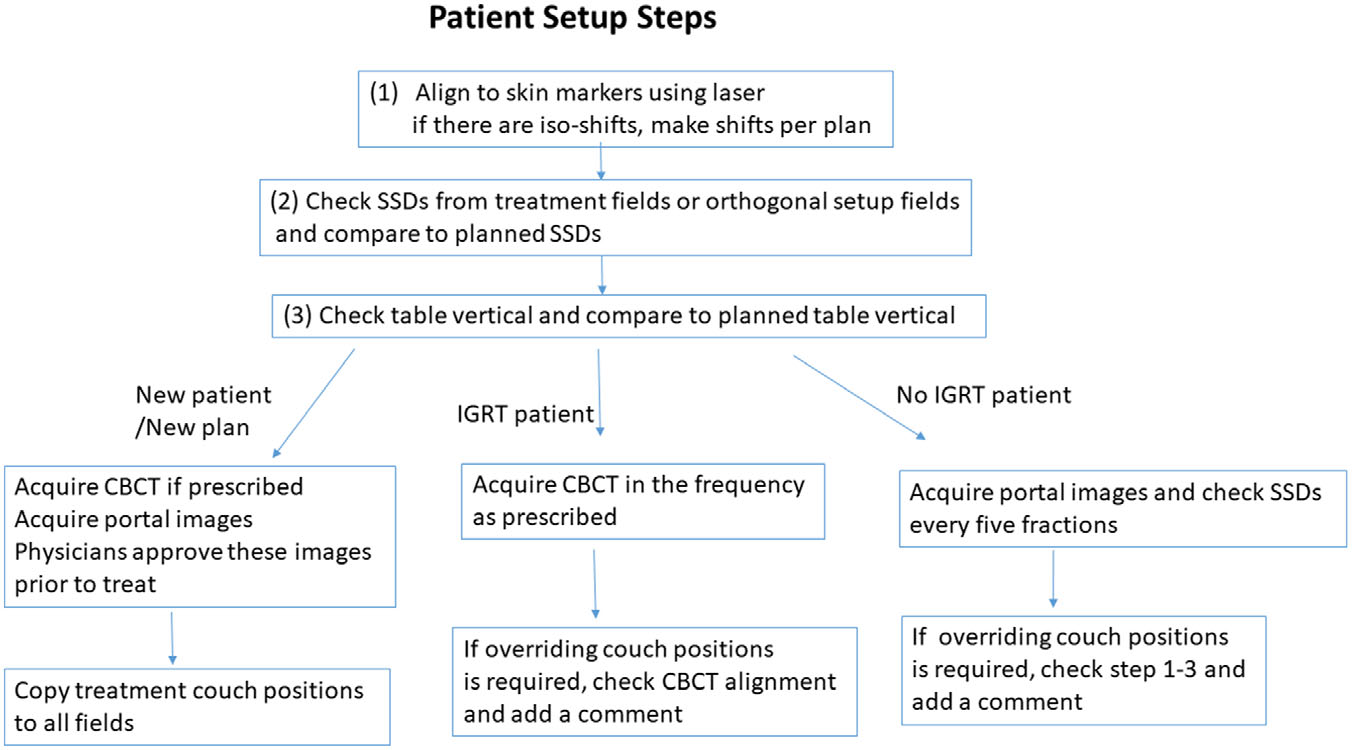
A flowchart of patient setup steps for new patients, patients with and without IGRT. IGRT is typically referred to kV‐CBCT. IGRT, imaging guided radiotherapy; kV‐CBCT, kilo‐voltage cone‐beam CT

Figure 1 illustrates our patient positioning verification procedure prior to each treatment. Depending on treatment modalities and treatment status (new vs. ongoing), procedures for patient position verification differ. The verification procedure for any new patient (defined as starting a new plan) depends on whether daily or other IGRT frequency is prescribed. For any new plan, whether three‐dimensional (3D) conformal or intensity‐modulated radiotherapy (IMRT), the skin‐to‐source distances (SSDs) for all treatment fields of 3D plans, and SSDs of orthogonal setup fields of IMRT plans are verified on the first day of treatment and weekly thereafter. The planned treatment couch vertical, determined to be a reliable patient positioning parameter, is measured during CT planning and also calculated by an in‐house script developed in the Pinnacle system (Philips, Netherlands). Typical treatment sites without IGRT included breast, advanced stage lung cancer, pelvis irradiation for rectal and gynecology cancers, palliative treatments, and treatments with only electron fields.

### 2.2 Quality controls of treatment delivery

**FIGURE 2:**
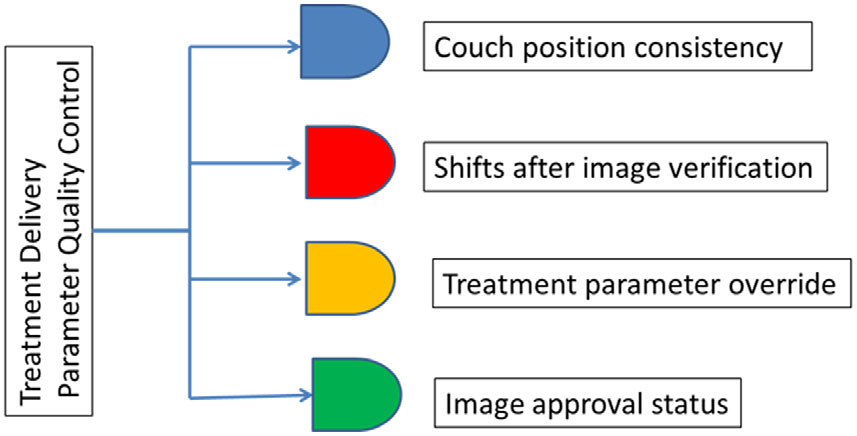
Four quality control metrics that measure quality controls of treatment delivery

Following the described treatment position verification procedure, we identified four QC metrics that can quantitatively measure QCs of treatment delivery (Figure 2). Treatment couch position consistency and overrides are the two most important metrics to control and monitor patient positioning precision. For stereotactic body radiotherapy (SBRT) treatments, both a physician and a physicist are present at the console area to approve image registration prior to each treatment delivery. For non‐SBRT treatments, depending on the specific treatment site, large shifts (>1.0–2.0 cm) made using imaging guidance are flagged for in‐room investigation. For new patients, as shown in Figure 1, physicians must approve all portal images and CBCTs (if prescribed) prior to delivery of the first treatment. For patients under treatment, all verification images (including orthogonal images and CBCT images) are approved by the attending physician on the day of treatment.

### 2.3 Treatment couch position tolerance tables

According to the treatment intent, modalities, and sites, we developed six treatment couch position tolerance tables listed in Table 2 and accompanied with clinical examples. The differences in these tolerance tables were made to accommodate various clinical situations. For example, for treatment sites in thorax and abdomen, we allow 3.0‐cm couch position variations in both the lateral and superior–inferior directions due to the difficulty in adjusting heavy patients. For SBRT treatments, we allow 2‐cm couch position variations in both the lateral and superior–inferior directions and 1.5 cm in the vertical direction, owning to soft‐tissue alignment and 6DoF couch corrections. The rotation on the pedestal is strictly controlled (<0.1°) because the axis of the pedestal rotation is in the middle of the treatment couch, not at the isocenter; a small rotation on the pedestal can cause a large degree of rotation at the treatment isocenter depending on the distance from the isocenter to the center of the couch. To validate our tolerance table settings for patient treatment under daily IGRT, we analyzed the couch shifts after kV‐CBCT for three treatment sites from 2018 to 2021.

**TABLE 2:** Couch position tolerance limits based on treatment sites

Name	Lateral (cm)	Longitudinal (cm)	Vertical (cm)	Angle	Pedestal	Clinical examples
HN	1.5	1.5	1.0			Brain immobilized with mask
HN‐CBCT	1.5	1.0	1.0	3	0.1	HN
Body	3.0	3.0	1.5	1.0	0.1	Breast, electron plans based on CT
Body‐CBCT	3.0	3.0	1.0	3	0.1	Thoracic, abdominal, and prostate cases
SBRT‐body	2.0	2.0	1.5	3	0.1	Lung, liver, spine Mets
Electron‐clinical setup	10.0	20.0	3.0			Clinical setup without CT planning

Abbreviations: HN‐CBCT, head and neck cone‐beam CT; SBRT, stereotactic body radiotherapy.

### 2.4 Incident reporting systems

We have two incident reporting systems. The first is our department's process improvement system (Workflow Enhancement—WE Forms), designed to report errors discovered during routine quality and safety check processes, any process deviations (such as patient chart arrival delays, scheduling errors resulting in delays), and unclear communications (such as incomplete simulation requests).^6^ The second is our hospital's incident reporting system that reports any deviation from the radiation prescription and the deviation reaches a patient (clinically significant or not). Such a deviation requires immediate attention and signatures by the attending physician, department chair, head of physics, manager of therapists, and quality coordinator. These incident reports include a detailed description of the incident, dosimetric evaluation, and a brief root cause analysis.

### 2.5 QMAP system

In addition to our incident reporting systems, we have developed a quantitative metric and automated auditing program (QMAP), described previously,^14^ to proactively control several key processes and thereby reduce the number of medical incidents and WE forms. In our previous report, we focused on timely completion of critical tasks to reduce process interruptions and distractions. In this study, we used the QMAP system to automatically produce a daily report that includes couch position overrides for all treatments delivered the previous day. These overrides were typically initiated by one of the treating therapists and corroborated by a second therapist. A brief description of the reason for any overrides was also required to serve as a timeout. The daily override reports were sent to the therapist in charge of and the physicist responsible for each treatment machine the following morning, allowing them to conduct a timely review and determine whether the comments and magnitudes of the overrides were appropriate. This study analyzed the treatment couch position overrides for all patients treated with external beam radiation at our main campus from January 2012 to December 2018.

### 2.6 Data collection

Our analysis included overrides in the lateral, longitudinal, and vertical couch positions. Overrides for the first day of treatment were excluded because only vertical couch positions were established prior to the first fraction, and lateral and longitudinal couch positions were not yet set. Therefore, overrides in these directions were expected. Also excluded were couch position overrides when, during a course of treatment, it was necessary to change Linacs, some of which were from different venders (having different characteristics) during the initial part of the study period.

## 3 RESULTS

**FIGURE 3:**
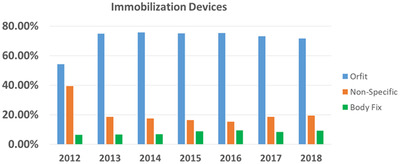
The frequency of immobilization devices used from 2012 to 2018

**FIGURE 4:**
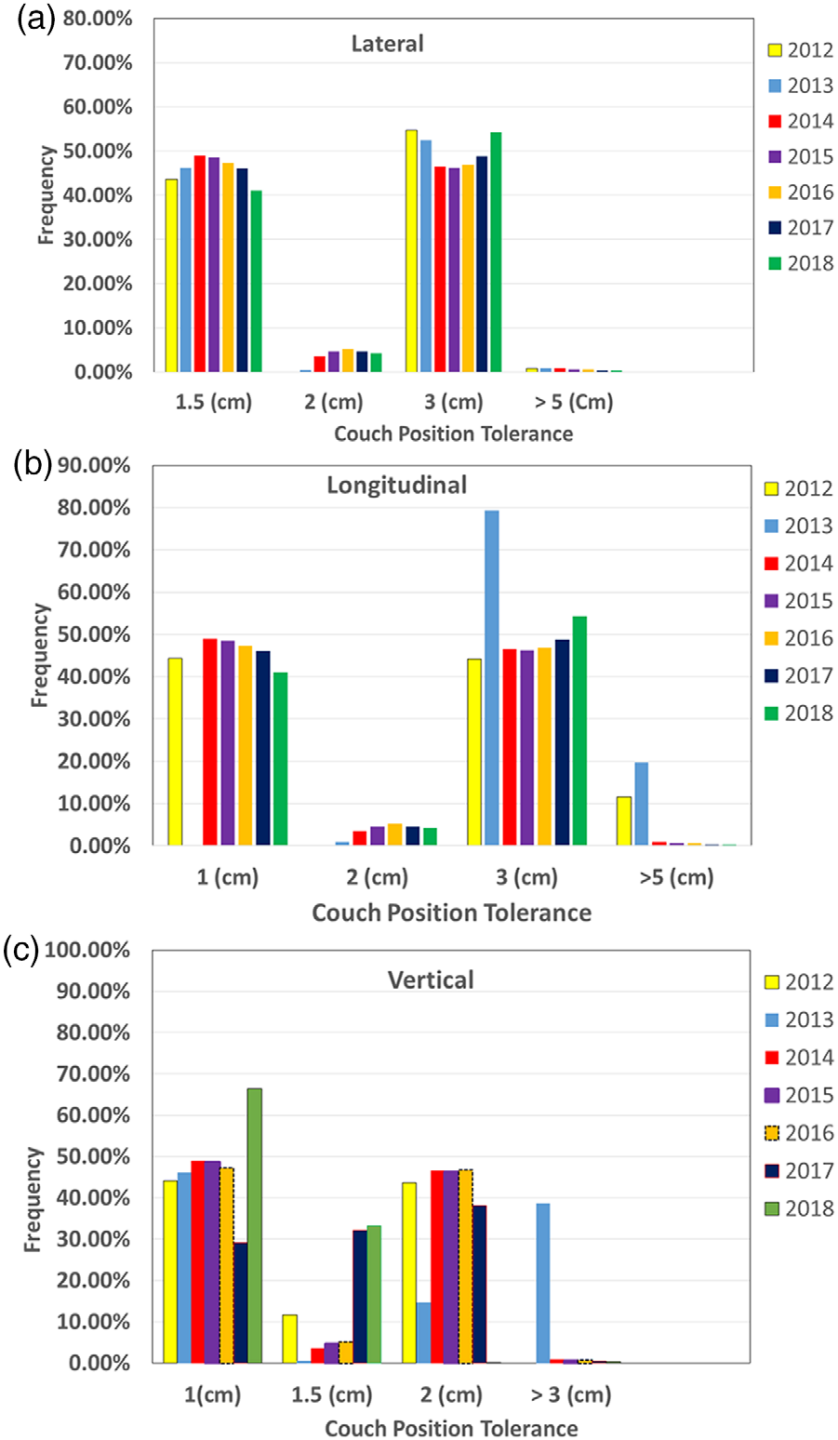
The ranges and frequencies of couch position tolerance limits (in cm) applied in the (a) lateral, (b) longitudinal, and (c) vertical directions from 2012 to 2018

Figure 3 shows the frequency of immobilization devices used in our department over the study period. More than 75% of the fractions were treated with the Orfit immobilization devices from 2013 to 2018. In 2013, we reduced the number of fractions by not using any immobilization devices (or not clearly specified in radiation oncology–specific electronic medical record), from 40% to less than 20%, which represented a typical number of patients treated in our department for urgent medical needs (most of them were inpatient). Figure 4a–c shows the ranges and frequencies of couch position tolerances applied in the lateral, longitudinal, and vertical directions from 2012 to 2018. Of note, the numbers in the *x*‐axis in Figure 4a–c are the couch position tolerance limits set for all patients over the period. As shown in Figure 4a, the pattern of applying couch limits in the lateral direction was consistent over time. Three of six tolerance tables (see Table 2) allowed variations of the lateral couch position within 3 cm. In the longitudinal direction, the frequencies of applying couch position limits ≥3 cm were reduced, especially after 2013. In the vertical direction, we gradually reduced the frequency of applying couch position limits >3.0 cm after 2013 and limits >1.5 cm in 2018.

**FIGURE 5:**
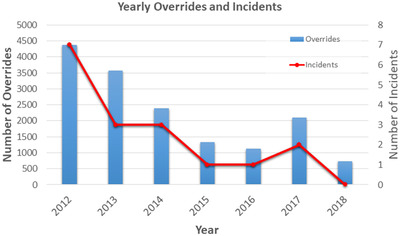
The annual number of overrides and the number of positioning errors from 2012 to 2018

Figure 5 shows the annual number of overrides and the number of positioning errors, reported in our hospital incident report system from 2012 to 2018. Root cause analyses of these positional errors pointed to careless couch positional overrides. None of the positioning errors caused medically significant harm to patients due to early error discovery. During the study period, the number of fractions treated at the main campus increased from 36 684 to 45 010 (23% increase), whereas the number of overrides decreased from 4374 (11.2% of all fractions) to 728 fractions (1.6% of all fractions). As shown in Figure 3, the use of immobilization devices remained constant from 2013 to 2018. In 2013, the use of Orfit immobilization devices increased from 54% to 75% of the total number of fractions. In 2017, our department replaced all the Linacs, equipping each new Linac with a treatment couch that had a different sag (which impacts on vertical couch position) than the treatment couches of the replaced Linacs. Consequently, the introduction of a new treatment couch initially resulted in a spike (see Figure 5) in the number of overrides, particularly in the vertical direction. A systematic couch position correction of 0.5 cm was introduced into the planned couch vertical position to offset the specific couch sag. Since then, the downward trend of overrides has continued, reaching the recorded low number of 728 (1.6% of all fractions) in 2018. As shown in Figure 5, the annual patient positioning errors were decreased as a function of the number of overrides. Because of the extremely small number of patient position errors, any correlation calculation would not be meaningful, but a linear decrease trend was observed (*R* ^2^ = 0.87). The patient positioning error rate was 0.019% in 2012, decreased to 0.004% in 2017, and 0% in 2018.

**FIGURE 6:**
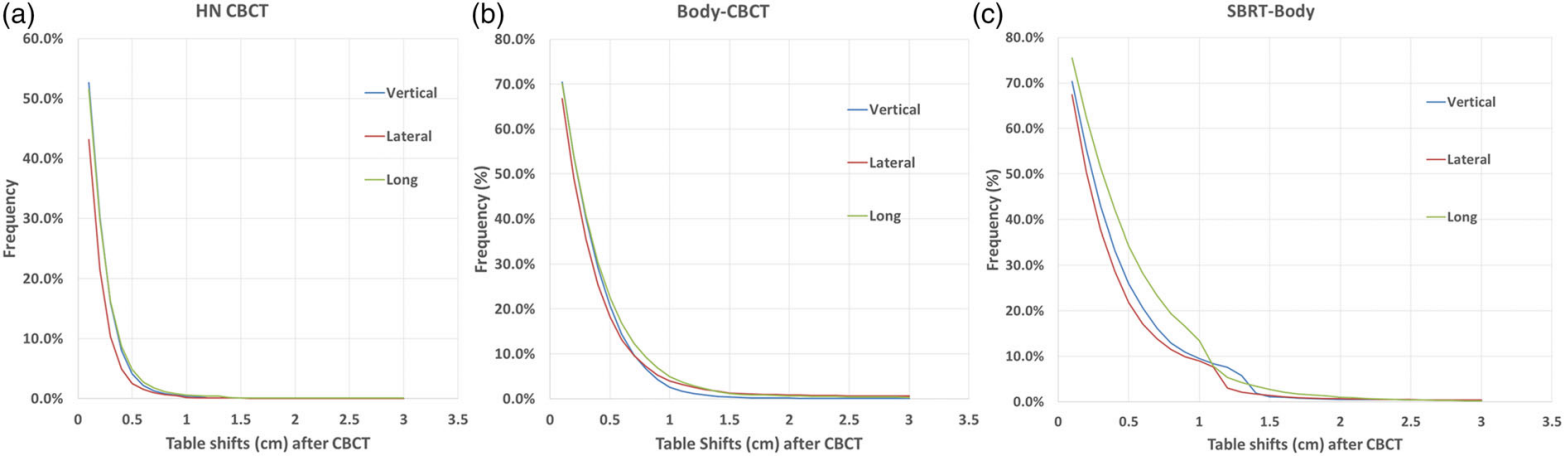
The recorded table shifts after kV‐CBCT verifications for the treatment site of (a) HN‐CBCT; (b) body‐CBCT; (c) SBRT‐body, where the corresponding couch position tolerance was applied. HN‐CBCT, head and neck cone‐beam CT; kV‐CBCT, kilovoltage cone‐beam CT; SBRT, stereotactic body radiotherapy

Figure 6a–c shows the recorded table shifts after kV‐CBCT verifications for three treatment sites where tolerance tables of head‐and‐neck CBCT (HN‐CBCT), body‐CBCT, and SBRT‐body were applied. Figure 6a–c shows that after kV‐CBCT, the applied couch shifts that exceeded the couch position limits were low, <1% for HN‐CBCT, <1.2% for body‐CBCT, and <2.6% for SBRT‐body. Figure 6a shows that for patients with HN‐cancer and brain tumors, the table shifts after CBCT verification in lateral, vertical, and longitudinal directions were <0.5 cm in 95% of treatment fractions, indicating that our patient positioning tolerance limits are adequate and perhaps can be further reduced. These patients were typically immobilized with masks and set up on an indexed treatment couch. For patients treated with IMRT for lung cancer, abdomen cancer, and pelvis cancer, the tolerance table of body‐CBCT was frequently applied. Figure 6b shows that the table shifts after CBCT verifications in the lateral, vertical, and longitudinal directions were <1.0 cm in >95% of treatment fractions, also indicating that our tolerance limits for this group of patients are adequate and perhaps can be reduced. For patients treated with SBRT for lung cancer, abdomen cancer, and pelvis cancer, the tolerance table for SBRT‐body was frequently applied. Figure 6c shows that the table shifts after CBCT verifications in the lateral, vertical, and longitudinal directions were <1.2 cm in >95% of fractions. Of note, for SBRT patients, the CBCT alignments were frequently aligned to tumors, not bony structures, explaining relative larger shifts than the shifts from patients in the body‐CBCT group.

## 4 DISCUSSION

We demonstrate that controlling treatment couch position overrides is a very important QC measure in treatment delivery, noting that in the last 7 years, the treatment couch position override rate decreased with a concomitant decrease in treatment positioning errors. Our experience shows that a timely review of the overrides is effective in enhancing quality and safety. As the treatment couch positions are surrogates for patient positioning, controlling tolerance limits on the treatment couch positions for each patient is not trivial. The novelty of the study was the use of daily monitoring and automatic reports to enforce cautious table position overrides. Hadley et al. noted that a tight tolerance limit may increase overrides and cause override fatigue, whereas a loose tolerance limit may defeat the purpose of setting a couch position limit, rendering it ineffective.^15^ Under daily image guidance, one may think that it is not important to control the consistency of couch positioning. Our clinical experience indicated that even with daily imaging guidance, risks of accidentally dragging an aligned CBCT image off after image registration along with risks of wrong image registrations make the image guidance not entirely error‐proof. Setting a reasonable couch position limits and enforcing cautious overrides can prevent potential positional errors.

After reviewing table shifts for patients treated with CBCT verifications, we confirmed that our tolerance tables are adequate and perhaps can be further reduced. We decided to merge the tolerance tables of body‐CBCT and SBRT‐body into one tolerance table and set the tolerance limit to 1.5 cm in each direction. Under these new tolerance limits, the vertical of body‐CBCT increased from 1.0 to 1.5 cm taking into consideration that more patients are treated on 6DoF couches. Similarly, because most of our patients with HN‐cancer and brain tumors are treated under daily IGRT on 6DoF couches, we decided to keep couch position limits of HN‐IGRT unchanged, although the data from Figure 6a indicated that we can reduce these limits to 0.5 cm.

Over the years, we have taken the following steps to reduce treatment couch position overrides. First, we require therapists to document any overrides performed and have a second therapist to corroborate that the overrides occurred. For any overrides >2.0 cm outside of tolerance limits, we require therapists to perform an in‐room investigation. For SBRT treatment, our policy is to have a physician and a physicist to approve the image registration prior to any treatment delivery. Based on our analysis, the most effective action to reduce overrides is the daily report of overrides from the prior day's treatment. This daily report is reviewed by the therapist in charge of the treatment machine, the physicist responsible for the treatment machine, and the dosimetrists for the specific treatment site. We believe that a punctual review of overrides allows us to detect errors early in the treatment course, preventing a significant medical incident. Furthermore, we list the daily override data on a website to assist physicists to review overrides during the weekly chart check.

The topic of how to set the tolerance limits has been discussed by others. Assuming that couch position is a random variable and the ideal planned couch position is unknown, Hadley et al.^15^ compared the first day acquired couch position as the baseline with the cumulative average couch position as the baseline, concluding that using the cumulative average couch position as the baseline increased the sensitivity of detecting out‐of‐tolerance positions. McCullough et al. created their treatment couch tolerance tables based on a retrospective analysis of 66 patients treated in 1308 fractions and then validated the set tolerance tables for an additional 65 patients treated in 1504 fractions.^16^ They found that with couch baseline values updated with every image fraction, the override rate was 10%, and without baseline updating (using the first day treatment as a constant baseline), the override rate was 16%. Chinsky et al. surveyed table tolerance and couch overrides within American Association of Physicists in Medicine (AAPM).^17^ In Table 3, comparing our couch tolerance limits, we listed the couch tolerance limits from the AAPM survey result, the publication from the University of Michigan,^15^ and the publication from the University of Louisville.^16^ Except for the vertical tolerance limits, our tolerance limits on the lateral and longitudinal directions for HN/brain sites are less stringent than the limits from the other three tolerance tables. Our tolerance limits for body without CBCT guidance are similar to the published results. With the availability of 6DoF tables in our department, we allow 3° limit for HN‐CBCT, body‐CBCT, and SBRT‐CBCT because these sites are often treated with 6DoF corrections. The SBRT‐body tolerance table only applies to all SBRT sites except for brain SRS, as we use Gamma Knife to treat all brain SRS cases. For SBRT lung and liver, we typically align to the tumor or soft tissues, therefore, purposely set tolerance limits to reflect this practice. In our department, simulation CTs are acquired for all photon plans and most of electron plans. For a small number of patients, their treatment plans with electron beams may be based on the clinical setup, for which the light field is used to visualize the treated area. Therefore, our limits on the electron tolerance table for clinical setup are very loose when compared to the AAPM survey result.

**TABLE 3:** Comparison of our tolerance tables and proposed new tolerance tables (in parenthesis) with three other published tolerance tables

	Lateral (cm)				Longitudinal (cm)				Vertical (cm)			
Name	CCF	UL^a^	UM^b^	Survey^c^	CCF	UL	UM	Survey	CCF	UL	UM	Survey
HN	1.5		0.6	1	1.5	0.7	1		1.0		0.7	1
HN‐CBCT	1.5	0.9	0.9	1	1.0	0.9	0.9	1	1.0	0.5	0.7	1
Body	3.0	3.5	2.7	>5	3.0	3.8	2.5	>5	1.5	0.9	0.7	1
Body‐CBCT	3.0 (1.5)		>5		3.0 (1.5)			>5	1.0 (1.5)			1
SBRT‐body	2.0 (1.5)			0.3	2.0 (1.5)		0.3		1.5 (1.5)			0.3

Abbreviation: CCF, cleveland clinic foundation; HN‐CBCT, head and neck cone‐beam CT; SBRT, stereotactic body radiotherapy; UL, University of Louisville; UM, University of Michigan.

^a^ See Ref. [15].

^b^ See Ref. [16].

^c^ See Ref. [17].

Similar to our daily override report, Xia et al. described a comprehensive computer‐aided treatment event recognition system by analyzing electronic treatment records.^18^ They identified that couch position overrides, extra CBCT imaging, and significant couch position deviations were the top three frequently detected aberrant treatment parameters. Instead of controlling couch position, using surface imaging to setup patients has been introduced in recent years.^19^ Surface imaging allows for both patient positions and postures to be verified during the initial patient setup. This method is particularly useful for breast treatment setup as the postural changes, particularly arm positions, can significantly impact on the treatment target localization.^20^ Surface imaging is not available in every treatment room secondary to additional costs, including the installation of a three‐camera system. Further research is needed to investigate whether surface imaging will replace the current ubiquitous laser system.

## 5 CONCLUSION

Using a daily monitoring system enables a timely review of overrides, significantly reduces treatment couch position overrides, and ultimately decreases treatment positioning errors. As radiation oncology moves from the conventional equipment‐centered QA paradigm to patient‐ and process‐centered QA, controlling treatment positioning will be a part of patient‐centered QA metrics. A timely review of couch position overrides, as a component of our QMAP, is an effective process to augment index patient positioning.

## CONFLICT OF INTEREST

No conflict interest is related to this manuscript.

## AUTHOR CONTRIBUTIONS

Concept development: Naichang Yu, Ping Xia. Data analysis: Danielle LaHurd, Anthony Magnelli. Process refinements: Naichang Yu, Eric Murray, Mike Close, Brian Hugebeck, John H. Suh, Ping Xia. Manuscript writing: Naichang Yu, Ping Xia, Anthony Mastroianni, Danielle LaHurd.
